# Longitudinal assessment of SARS-CoV-2 IgG seroconversionamong front-line healthcare workers during the first wave of the Covid-19 pandemic at a tertiary-care hospital in Chile

**DOI:** 10.1186/s12879-021-06208-2

**Published:** 2021-05-26

**Authors:** Mirentxu Iruretagoyena, Macarena R. Vial, Maria Spencer-Sandino, Pablo Gaete, Anne Peters, Iris Delgado, Inia Perez, Claudia Calderon, Lorena Porte, Paulette Legarraga, Alicia Anderson, Ximena Aguilera, Pablo Vial, Thomas Weitzel, Jose M. Munita

**Affiliations:** 1 Laboratorio Clínico, Clínica Alemana de Santiago, Santiago, Chile; 2 Facultad de Medicina Clínica Alemana, Universidad del Desarrollo (CAS-UDD), Santiago, Chile; 3 Instituto de Ciencias e Innovación en Medicina (ICIM), Facultad de Medicina CAS-UDD, Santiago, Chile; 4 Millennium Initiative for Collaborative Research on Bacterial Resistance (MICROB-R), Santiago, Chile

**Keywords:** Covid-19, SARS-CoV-2, Seroprevalence, Seroconversion, Healthcare workers

## Abstract

**Background:**

Healthcare workers (HCWs) are at high risk of exposure to SARS-CoV-2. Cross-sectional studies have provided variable rates of seroprevalence in HCWs. Longitudinal assessments of the serological response to Covid-19 among HCWs are crucial to understanding the risk of infection and changes in antibody titers over time. We aimed to investigate seroprevalence and risk factors associated with seroconversion in a prospective cohort of HCWs during the peak of the first wave of the Covid-19 pandemic.

**Methods:**

We conducted a longitudinal study among 446 front-line HCWsin a tertiary-care hospital in Chile from April to July 2020. IgG was determined monthly using two different ELISAs in serum samples of HCWs, during the three-month period. In each visit, demographic data, symptoms, risk factors, and exposure risks were also assessed.

**Results:**

The overall seroprevalence at the end of the study period was 24% (95% CI20.2–28.3), with 43% of seropositive HCWs reporting no prior symptoms. Seroconversion rates significantly differed over the study period, from 2.1% to as high as 8.8% at the peak of the epidemic. There were no statistically significant differences observed between HCWs in direct clinical care of patients with Covid-19 and those working in low risk areas. Antibody titers appeared to wane over time.

**Conclusions:**

HCWs were severely affected with a high rate of seroconversion that appeared to mirror the local epidemiological situation. A significant amount of participants underwent an asymptomatic infection, highlighting the need for improved surveillance policies. Antibody titers appear to wane over time; further studies to understand this finding’s impact on the risk of reinfection are warranted.

**Supplementary Information:**

The online version contains supplementary material available at 10.1186/s12879-021-06208-2.

## Background

As of December 2020, Chile is among the most affected countries by the SARS-CoV-2 pandemic worldwide, with an overall incidence rate of > 33,957 cases of Covid-19 per 1 million population [1]. Infection of health care workers (HCWs) during an epidemic carries significant consequences both individually and at the community level as they may represent a source of transmission within the healthcare environment and elsewhere [2]. Moreover, high infection rates among HCWs increase workplace absenteeism, overloading the already highly burdened healthcare system. Although front-line healthcare personnel (i.e.*,* HCWs directly caring for Covid-19 patients) are at risk of occupational exposure with SARS-CoV-2 [3–6], the magnitude of this risk as compared to non-front-line HCWs and the general population is unclear. Previous seroprevalence studies have estimated variable rates of SARS-CoV-2 IgG seropositivity among HCWs, with data ranging from 4 to > 40% [7–9]. Of note, most of these efforts are cross-sectional evaluations (seroprevalence), and longitudinal assessments of the seroconversion rate of HCWs are limited to European centers [10–13]. Notably, such longitudinal assessments are crucial to better understand the dynamics of Covid-19 among exposed hospital personnel and help optimize local policies regarding infection surveillance, as exposure and employee behavior may change as the pandemic progresses. Moreover, understanding the kinetics of antibody titers may help design preventive measures and vaccination strategies for front-line HCWs. Here, we present a three-month longitudinal follow-up study of the SARS-CoV-2 IgG seropositivity and epidemiological features of front-line HCWs in a tertiary-care hospital during the peak of the first wave of the Covid-19 outbreak in Santiago, Chile.

## Methods

### Study setting

**Fig. 1:**
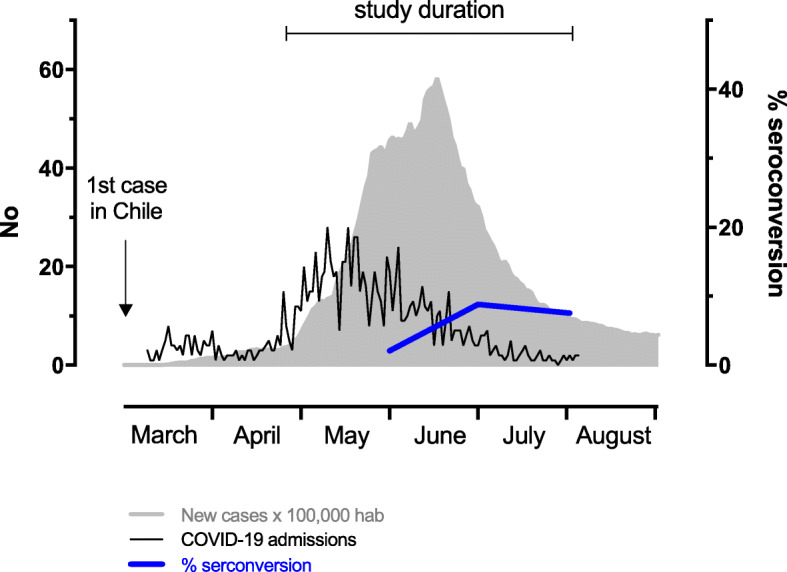
Regional trends of Covid-19 cases and rates of seroconversion among HCWs. The grey area represents the daily incidence rates per 100,000 inhabitants in Chile’s Metropolitan Region, determined by RT-PCR [14]. The black line shows the number of daily Covid-19 patient admissions at Clínica Alemana de Santiago (CAS). Monthly seroconversion rates of HCWs are represented with a blue line

The study was conducted in Clínica Alemana de Santiago (CAS), a not-for-profit private tertiary care hospital with > 440 beds and 1414 employees, located in the Metropolitan region, Santiago, Chile. The hospital was confronted with Covid-19 since the beginning of the country’s pandemic. The first patient was attended on March 4th, 1 day after the first case was diagnosed in Chile (Fig. 1) [15]. Shortly after, dedicated Covid-19 clinical areas were assigned, and a separate respiratory emergency room (ER) was organized for patients with possible SARS-CoV-2 infection. Mandatory use of personal protective equipment (PPE) (facemask, face shield, isolation gown, gloves) was required for HCWs in contact with suspected or confirmed Covid-19 cases. All hospital personnel were required to use surgical masks at all times since March 16th. Training on the correct use of PPE was performed to all personnel, and periodically reinforced. All staff members with confirmed Covid-19 or close unprotected contact with a confirmed case were quarantined for 14 days, as per national regulations. Daily incidence rates in the Metropolitan Region of Santiago and daily admission rates of Covid-19 patients at CAS are presented in Fig. 1.

### Study design and population

We performed a prospective longitudinal study among HCWs at CAS from April 27th to July 31st, 2020. The primary outcomes were IgG seroprevalence and IgG seroconversion to SARS-CoV-2. All front-line HCWs serving in clinical areas exclusively dedicated to Covid-19 patients (medical wards, intensive care units [ICUs], step down units, and respiratory ER) were invited to participate as “high-risk” group. In addition, HCWs from selected areas without direct contact with Covid-19 patients (i.e.*,*cardiothoracic ICU) were offered to participate as part of a “low-risk” group. Both groups included physicians, nurses, respiratory therapists, technicians, and paramedics. If participants changed from a low-risk to a high-risk setting, they were considered part of the high-risk group. HCWs with active symptoms or on quarantine as per national regulations were only enrolled once the mandatory isolation period was completed.

### Serum samples and data collection

Venous blood samples were collected at four time points: at study entry and monthly for 3 months (every 3–4 weeks for a period of 4 months, with a minimum interval of 15 days between samples). The serum was separated, aliquoted, and stored at − 20 °C until analysis. A standardized questionnaire was completed at each serum sampling appointment, including demographic data, occupation, clinical unit, comorbidities, exposure risks, and symptoms during the previous 4 weeks. Information regarding the history of quarantine and RT-PCR testing was obtained from the clinical laboratory and institutional infection control registries. Samples were analyzed anonymously in the laboratory and results were sent to study nurses who contacted each participant to report their results.

SARS-CoV-2 serology. Specific IgG was determined using two commercial enzyme-linked immunosorbent assays (ELISA): (1) Covid-19 ELISA IgG (Vircell, Granada, Spain) using recombinant SARS-CoV-2 nucleocapsid protein (N) and spike glycoprotein (S), and (2) Anti-SARS-CoV-2 ELISA IgG (Euroimmun, Lübeck, Germany) based on recombinant S1 domain of the S protein including the receptor-binding domain (RBD). We previously validated the assays in our laboratory using serum samples from 60 RT-PCR confirmed Covid-19 patients and 40 asymptomatic RT-PCR negative patients. Vircell assay had a 85.7% sensitivity and 98.5% specificity after 3 weeks of the initial symptoms. The Euroimmun IgG ELISA had a 80% sensitivity and a 100% specificity after 3 weeks of the first symptoms. All samples were screened with the Vircell assay, and samples positive with this test were confirmed using the ELISA kit from Euroimmun, which has a higher specificity [16]. Both tests were performed according to manufacturers’ instructions. Only samples reactive in both tests were considered seropositive. Seroconversion was defined as the change from a negative to a positive IgG result in subsequent samples. For IgG values, the sample’s optical density (OD) is divided by the OD of the cutoff control for each assay, as recommended by the manufacturer. To assess the dynamics of SARS-CoV-2 IgG levels over time, we selected subjects with a positive SARS-CoV-2 RT-PCR, > 2 follow-up serum samples, and a follow-up period of > 60 days after molecular testing. The samples of 10 subjects fulfilling these criteria were simultaneously re-tested using the Euroimmun assay.

### Statistical analysis

Data were collected and managed using REDCap electronic data capture tools hosted at Clinica Alemana. REDCap (Research Electronic Data Capture) is a secure, web-based software platform designed to support data capture for research studies [17]. All statistical analyses were performed using SPSS 22.0 and GraphPad prism9. Categorical variables are presented as frequencies and percentages. Comparisons between groups were performed using Chi-square test or ANOVA. Continuous variables are presented as mean and 95% CI, comparison was done using Student s t test. Univariate and multivariate Cox regression analysis were used to evaluate the odds of seroconverting during the study. Differences with *p* values of < 0.05 were considered statistically significant.

## Results

### General characteristics of the cohort

A total of 446 HCWs were included, with a median age of 39 years (IQR 21–67);324 (72.6%) were women. The cohort included HCWs from different clinical units and occupations (Table 1). Comorbidities were reported by 190 HCWs (42.6%), most frequently smoking (20.4%), obesity (7%), and hypertension (6.5%). Of all participants, 412 (92.4%) belonged to the high-risk and 34 (7.6%) to the low-risk group (29 participants changed from low risk to high risk group during the study period). Characteristics of both groups are described in Table S1. A total of 1561 serum samples were collected. Follow-up samples were available from 417 (93.5%) of participants; 286 (64.1%) provided the complete three follow-up specimens, while two and one follow-up samples were available in 99 (22.2%) and 32 (7.2%) of HCWs, respectively (Figure S1).

**Table 1:** Overall seroprevalence and seroconversion among healthcare workers

Variables	Overall seroprevalence			3-month seroconversion		
	n	%	95% CI	n	%	95% CI
Total	446	24.0	20.2–28.3	374	17.1	13.5–21.4
Gender						
Female	324	25.9	21.3–31.1	268	18.6	14.3–24.0
Male	122	18.9	12.6–27.2	106	13.2	7.7–21.5
Age groups (years)						
20–34	174	27.6	22.3–34.9	138	21.0	14.7–28.9
35–49	201	22.4	17.1–29.2	172	15.1	10.3–21.6
50–65	67	20.9	13.2–31.8	60	15.0	7.5–27.1
> 65	4	0	0.0–49.0	4	0	0.0–49.0
Work place						
High risk	412	24.3	20.2–29.1	345	17.9	14.1–22.5
Intensive care unit	88	21.6	14.3–31.2	75	14.6	7.6–24.3
Stepdown unit	90	32.2	23.4–41.8	71	25.3	15.9–36.8
Medical ward	103	29.1	21.3–39.1	81	24.7	17.2–34.9
Emergency department	118	16.1	11.2–24.6	109	11.9	6.8–19.9
Low risk	34	20.6	10.1–37.2	29	6.9	1.2–24.2
Profession						
Physician	163	22.1	16.2–29.3	145	16.6	11.3–23.1
Respiratory Therapist	24	16.7	7.4–35.1	23	17.4	7.5–37.3
Nurse	139	28.8	22.5–37.6	117	19.7	13.2–28.9
Technician/paramedic	115	23.5	17.2–32.7	85	15.3	9.5–24.6
Administrative worker	5	0	0.0–43.5	4	0	0.0–49.0

### SARS-CoV-2 IgG seroprevalence and seroconversion

**Fig. 2:**
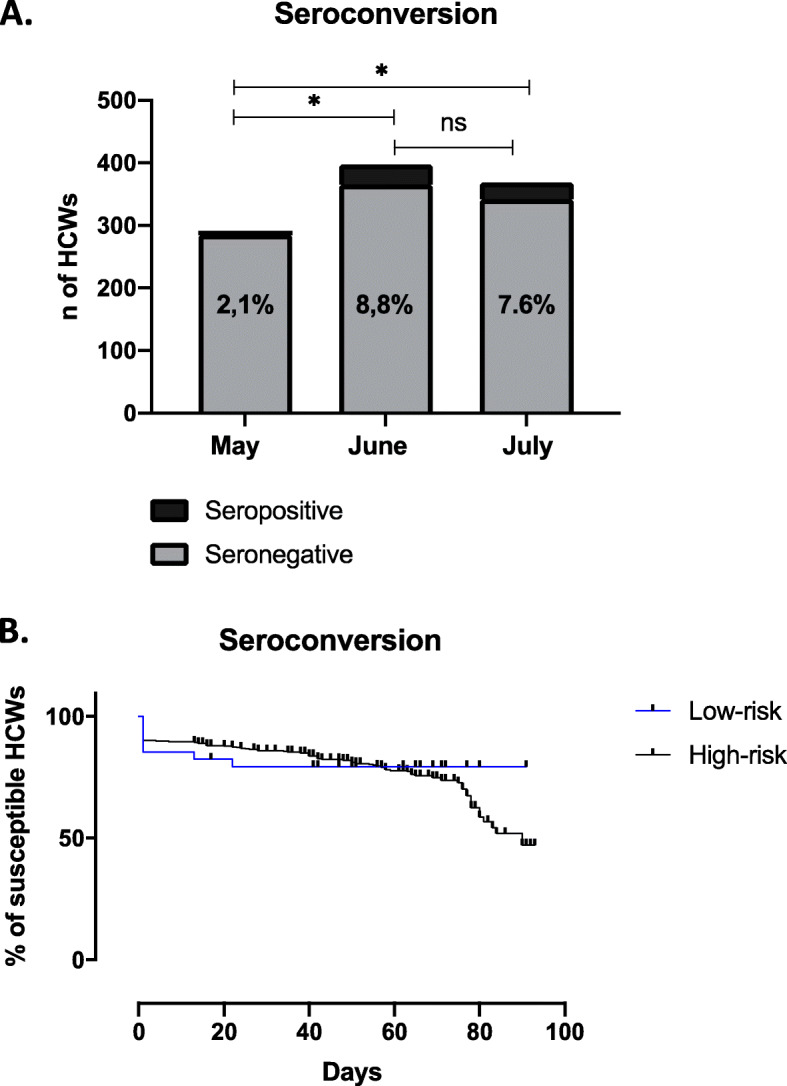
**A** Monthly seroconversion rates among HCWs (* *p* < 0.05, ANOVA) **B** Percentage of susceptible HCWs, comparing the high vs. low-risk groups (Mantel-Cox test, *p = 0.66*)

The overall seroprevalence, defined as SARS-CoV-2 seropositivity in at least one serum sample during the study period, was 107/446, 24% (95% CI 20.2–28.3). In the first evaluation, 43/446 (9.6%) HCWs were seropositive. Among the 374 initially seronegative participants who provided at least one follow-up sample (29 were lost to follow-up after the first sample), 64 (17.1, 95% CI 13.5–21.4) seroconverted during the study period (Table 1). Seroconversion rates significantly differed over the study period, with a lower rate in the first month (2.1, 95% CI 0.97–4.52) as compared to the second (8.8, 95% CI 6.28–12.12) and third months, respectively (7.6, 95% CI 5.24–10.9) (Figs. 1 and 2A). Neither seroprevalence nor seroconversion rates differed significantly when stratifying by gender, healthcare occupation, or clinical unit (Table 1). The overall seroprevalence and seroconversion rates of the high-risk group were higher than in the low-risk population (Fig. 2B); however, these differences did not reach statistical significance (24.3% vs. 20.6 and 17.9% vs. 6.9%, respectively; *p* > 0.05).

Among the 107 participants that tested IgG-positive at any time during the study, 86 (80.4%) reported prior testing with SARS-CoV-2 RT-PCR; of them, 75 (70.1%) were RT-PCR positive and 11 (10.3%) RT-PCR negative. Nine seronegative HCWs had a previous positive RT-PCR result; however, in eight of them, the time-interval between the positive RT-PCR and serological assessment was shorter than 3 weeks. The remaining subject, who suffered symptomatic Covid-19, remained serologically negative at days 19, 40, and 70 after the RT-PCR.

### Analysis of associated factors

The seropositive HCWs (*n* = 107) characteristics were compared to those remaining seronegative during the study (Table S2). The proportion of active smokers was significantly lower in seropositive than seronegative HCWs (11.2% vs. 23.3%;*p* = 0.004) (Table S2). In contrast, international travel in the previous 3 months and history of at least one Covid-19-compatible symptoms were associated with seropositivity (37.4% vs. 26%;*p* = 0.023 and 57% vs. 38.1%; *p* = 0.001, respectively) (Table S2). Importantly, 46 of 107 (43%) seropositive HCWs did not report any symptom compatible with Covid-19. Details of reported symptoms are provided in Table S2.

The multivariate Cox regression to identify factors associated with seroconversion demonstrated that diabetes (HR 17.4, 95%CI 3.9–77.8; *p* < 0.001) and a history of fever (HR 7.5, 95%CI 2.2–25.3; *p* = 0.001) or anosmia/ageusia (HR 6.7, 95%CI 3.8–11.5; *p* < 0.001) in the previous 4 weeks were independently associated with seroconversion (Table 2). In contrast, active smoking (HR 0.38, 95% CI 0.16–0.93; *p* = 0.03) and older age (HR 0.97, 95%CI 0.94–0.99; *p* = 0.03) were associated with a lower risk of seroconversion (Table 2).

**Table 2:** Cox regression model of variables related to seroconversion (person/days)

Variables	Univariate		Multivariate	
	HR (95%CI)	***p*** value	HR (95%CI)	***p*** value
Gender; Female	1.45 (0.80–2.63)	0.22		
Older Age	0.97 (0.95–0.99)	0.04	0.97 (0.94–0.99)	0.03
Work area High-risk for Covid-19 Low-risk for Covid-19	2.45 (0.59–10.03)	0.21		
Comorbidities & medications				
Diabetes	7.04 (1.68–29.61)	0.008	17.42 (3.9–77.83)	0.00
Hypertension	0.44 (0.11–1.82)	0.26		
Obesity	0.61 (0.19–1.96)	0.42		
Asthma	0.98 (0.24–4.00)	0.98		
Smoker	0.38 (0.17–0.89)	0.025	0.38 (0.16–0.93)	0.03
No comorbidities	0.78 (0.48–1.28)	0.33		
Use of ACE inhibitors	0.67 (0.21–2.14)	0.50		
Profession				
Physician	0.89 (0.54–1.48)	0.66		
Respiratory therapist	0.91 (0.33–2.51)	0.86		
Nurse	1.35 (0.81–2.25)	0.26		
Technician/paramedic	0.87 (0.47–1.60)	0.65		
Administrative worker	0.05 (0.0–6078.3)	0.61		
Clinical Unit				
Intensive care unit	0.78 (0.41–1.49)	0.46		
Stepdown unit	1.37 (0.79–2.37)	0.26		
Medical ward	1.50 (0.88–2.55)	0.13		
Emergency department	0.60 (0.33–1.11)	0.10		
Epidemiological risk factors				
Non-occupational Covid-19 contact	1.41 (0.57–3.52)	0.46		
International travel (previous 3 months)	1.82 (1.11–2.99)	0.018	1.59 (0.95–2.68)	0.08
Covid-19 related symptoms within 4 weeks of serology testing				
No symptoms	0.88 (0.53–1.45)	0.61		
Fever	3.43 (1.07–11.02)	0.04	7.54 (2.24–25.34)	0.001
Cough	1.47 (0.75–2.88)	0.27		
Myalgia	0.96 (0.30–3.07)	0.96		
Anosmia or Ageusia	7.35 (4.47–12.07)	0.00	6.7 (3.89–11.52)	0.00
Chest pain	2.47 (1.06–5.74)	0.04	1.22 (0.49–3.05)	0.66

### Analysis of SARS-CoV-2 IgG levels

**Fig. 3:**
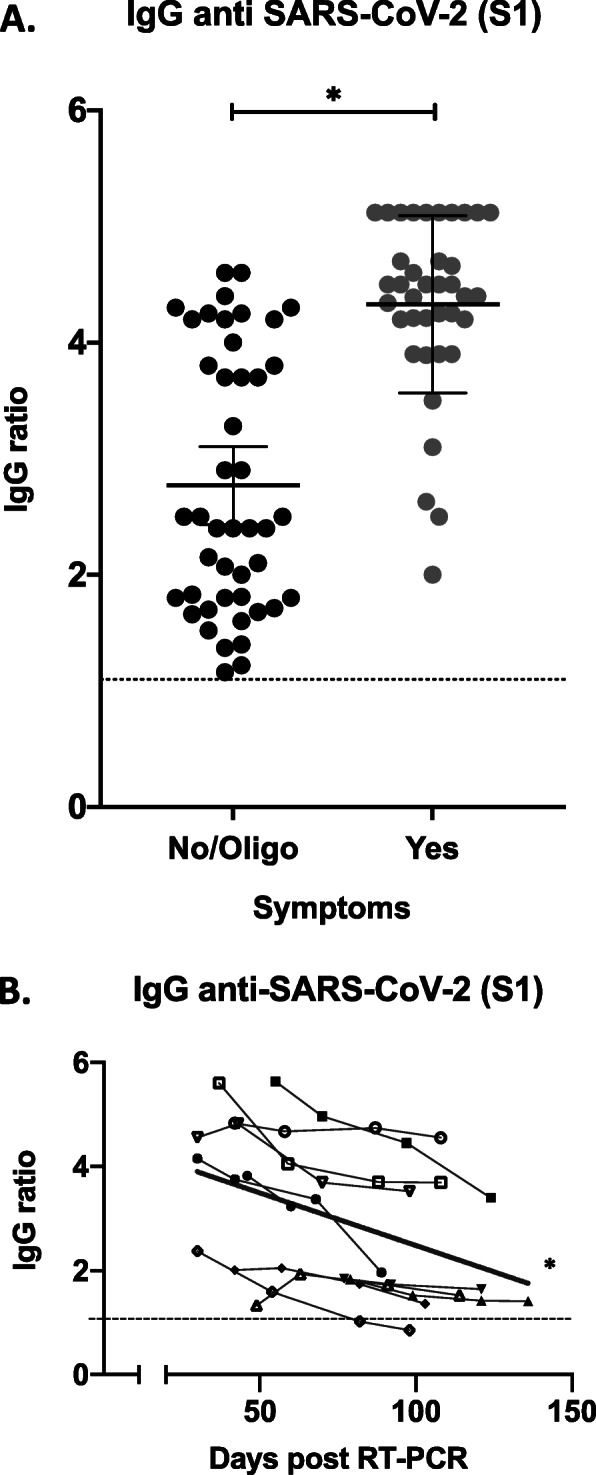
Analysis of IgG levels **A** Comparison of IgG ratios of asymptomatic/oligosymptomatic (No/oligo) and symptomatic participants (line and bars represent mean with 95% CI)(* *p* < 0.05, t-test). **B** Follow-up of IgG levels of 10 participants with proven Covid-19 (positive RT-PCR) during the study period (*Non linear reg, r^2^ 0.63)

The serum IgG levels were significantly higher among symptomatic participants than those remaining asymptomatic or oligosymptomatic (i.e., only one of the following symptoms: headache, coryza, or odynophagia) (Fig. 3A). In contrast, IgG values did not differ significantly between subjects with a previously positive RT-PCR and those with a negative or no previous RT-PCR (Figure S2).

In order to assess the variations of SARS-CoV-2 IgG levels over time in our study population, we selected samples from subjects with IgG measurements (> 2) at least 60 days after a positive RT-PCR (as described in methods). Simultaneous analysis of the samples fulfilling these criteria showed decreasing IgG levels during a maximum observation time of 140 days, seen in Fig. 3B. However, only one participant suffered a sufficiently marked decrease in titers to become seronegative at day 100 after the positive RT-PCR. We detected anti-spike antibodies up to 140 days after a positive molecular test.

## Discussion

Occupational infection of HCWs is one of the hallmarks of person-to-person transmission in communicable disease epidemics [18]. Although several studies have examined SARS-CoV-2 IgG seroprevalence among HCWs, to our knowledge, the present study provides the first data on Covid-19 exposure of HCWs in South America. We present a prospective cohort of HCWs with repeated assessments of SARS-CoV-2 IgG over a 3-month period, during the peak of the first wave, in a highly affected region in South America. In our longitudinal study, while the initial overall IgG seroprevalence was 9.6%, it reached a final cumulative value of 24%. In addition, IgG seroconversion appeared to mirror the local epidemiological situation, with values that increased from 2.1% during the first month of the study to 8.8% at the peak of the first epidemic wave.

In our study, the observed overall seroprevalence of 24% was high, compared to most previous studies, which mostly report rates of less than 15% [19–21]. However, reports from New York City (USA) and Birmingham (UK) showed similar rates, of 27 and 24.4%, respectively [7, 22]. Moreover, a recent meta-analysis reported a mean IgG seroprevalence among HCWs of 8.7%, ranging from 1.6% to as high as 44% in the UK [23, 24]. Of note, all studies included in the analysis derived from European and North American healthcare centers, and the situation in developing regions might differ.

One of the first studies providing longitudinal data was performed in Munich and reported seroconversion rates of 4.7% over a 3-month follow-up [11]. Similarly, a Spanish study performing a 1-month follow-up found seroconversion rates of 3.3% [10]. A study from Italy, shows that seroprevalence in HCWs in a referral hospital from Milan rose from 0.5 (95% CI 0.1 to 1.7) to 5.4 (95% CI 3.6 to 7.9) during the first month of the first wave [13]. Notably, our observed seroconversion rate of up to 8.8% during the pandemic first wave was much higher than in previous reports. These differences among studies may be explained by several factors that include: *i)* study design *ii)* timeliness and enforcement of infection control measures (e.g., routine screening, universal masking), *iii)* local epidemiological situation, *iv)* socioeconomic and cultural differences, and *v)*antibody testing assays and sampling strategies.

Direct clinical care of patients with Covid-19 was not associated with a higher risk of IgG seroconversion, nor was the clinical unit or the healthcare occupation. A lack of association between direct clinical exposure or clinical units and the risk of SARS-CoV-2 infection has been reported elsewhere [13, 20, 25–27]. However, it is worth considering that our study was underpowered to detect differences among subgroups. In addition, we did not measure essential variables such as compliance with infection control policies (e.g., hand washing, usage of personal protective equipment) and duration of direct exposure to Covid-19 patients. Our data, along with previous reports, raises the possibility that a significant proportion of HCWs acquire the infection in the community and not in the healthcare environment. While we did not find an association between close contact with a suspected or confirmed COVID-19 case outside the hospital and IgG seroconversion, this connection has been reported elsewhere [28], and most probably depends on the respective epidemiological and sociocultural settings. In our case it seems plausible, since Santiago was among the urban areas with highest COVID-19 incidences worldwide during our study period, and many HCWs live in districts distant from the hospital with long daily journey to and from work (including public transportation), having a high risk of exposure outside work. Moreover, Sikkema et al. recently demonstrated the introduction of SARS-CoV-2 from the community to the healthcare institutions using whole genome sequencing, further supporting this possibility [29]. In the setting of widespread community transmission, HCWs are at risk for community acquisition as well as potential work-related infection, and a combination of healthcare and community exposure likely contributes to seroprevalence.

Active smokers exhibited lower IgG seroconversion rates compared to the non-smoking group, findings that have also been previously reported [30–32]. This difference may reflect a decreased ability to mount an antibody response or a lower incidence of SARS-CoV-2 infection in active smokers. The hypothesis of lower infection rates in the smoking population has been previously explored, postulating that nicotine’s interaction with the acetylcholine receptor might impair SARS-CoV-2 cell entry [33]. Alternatively, the differences observed could be explained by social behaviors. For instance, given that smoking is widely forbidden within healthcare institutions, smokers are likely to spend more time in open environments. Recent behavioral data demonstrated HCWs staying in the same personnel break room with other co-workers exhibited a higher risk of SARS-CoV-2 infection, further supporting this hypothesis [34]. The analysis of factors associated with seropositivity showed a slightly increased hazard ratio for younger age, in line with other studies [6], and this factor can also be associated with less patient contact in the older HCWs. In the univariate and multivariate analysis, diabetes appears as an important risk factor for seroconversion, although significant, this observation is based only on 4 participants with diabetes, so this result needs to be further evaluated.

A prior history of Covid-19 symptoms was significantly more frequent in HCWs with a positive SARS-CoV-2 IgG result. As previously reported, anosmia and ageusia exhibited the strongest association with IgG positivity (HR 6.7; 95% CI3.89–11.52) [28]. It is noteworthy that 43% of seropositive HCWs did not report any symptoms. These data, along with previous studies, highlight the importance of including asymptomatic healthcare personnel in occupational surveillance strategies [27, 35, 36].

A rapid decrease in the IgG titers of HCWs has been recently reported, with levels reaching values below the positive threshold after an average follow-up of 137 days [37]. Similarly, our longitudinal analysis following participants for at least 100 days demonstrated an overall significant decrease in IgG titers. Despite this trend, titers decreased below the positive threshold in only one subject, but a more extended follow-up period may reveal a higher proportion of participants reaching that level. Importantly, although positive IgG titers have been associated with decreased risk of acquiring SARS-CoV-2 infection [38], the implications of waning antibodies are unclear and might not necessarily correlate with a higher risk of infection. Indeed, studies in SARS-CoV-1 demonstrated that, although specific IgG antibodies and memory B-cells were undetectable after a 6-year follow-up, specific T-cell anamnestic response was maintained in a significant proportion of cases [39]. When evaluating neutralizating antibodies, Harrington et al. recently demonstrated a rapid decline in these antibodies in patients recovered from Covid-19 [40]. Further studies with longer follow-up and ideally assessing the role of both cellular and humoral immunity are essential to developing optimal vaccine strategies.

Our study has some limitations that are worth mentioning. First, the reduced number of participants does not allow comparisons among sub-groups with appropriate statistical power. Second, loss to follow-up may have introduced selection bias; it is possible that more concerned HCWs (due to a high-risk behavior at work or elsewhere) were more likely to return for serology testing, resulting in an overestimation of seroconversion rates. However, it is reassuring that > 86% of participants provided at least two serum samples. In addition, one of the strengths of our strategy is the use of a two-tier serological diagnosis, increasing the specificity of our results.

## Conclusions

In conclusion, HCWs were severely affected with a high rate of seroconversion that appeared to mirror the local epidemiological situation. A significant number of participants underwent an asymptomatic infection, highlighting the need for improved surveillance policies. Further studies are needed to understand the impact of waning SARS-CoV-2 antibody titers on the risk of re-infection.

## Supplementary Information

**Additional file 1: Figure S1.** Study Flowchart.

**Additional file 2: Figure S2.** Comparison of IgG ratios of RT-PCR positive vs. negative participants (line and bars represent mean with 95% CI)(*p* = 0.09, t-test).

**Additional file 3: Table S1.** Demographic variables of high-risk and low-risk groups.

**Additional file 4: Table S2.** Characteristics of seropositive and seronegative healthcare workers.

**Additional file 5.** Participant Questionnaire.

## Data Availability

The datasets used and/or analyzed during the current study are available from the corresponding author on reasonable request.

## Abbreviations

HCWs: Healthcare workers
SARS-CoV-2: Severe acute respiratory syndrome coronavirus 2
Covid-19: Coronavirus disease 2019
CAS: Clínica Alemana de Santiago
PPE: Personal protective equipment
ER: Emergency room
ICU: Intensive care units
ELISA: Enzyme-linked immunosorbent assays
