# Supplementary figures and images for: Longitudinal assessment of SARS-CoV-2 IgG seroconversionamong front-line healthcare workers during the first wave of the Covid-19 pandemic at a tertiary-care hospital in Chile
Source: BMC Infect Dis. 2021 May 26;21:478. doi: 10.1186/s12879-021-06208-2 (PMC8149923; doi:10.1186/s12879-021-06208-2)

**FIGURE S1. Study flowchart**

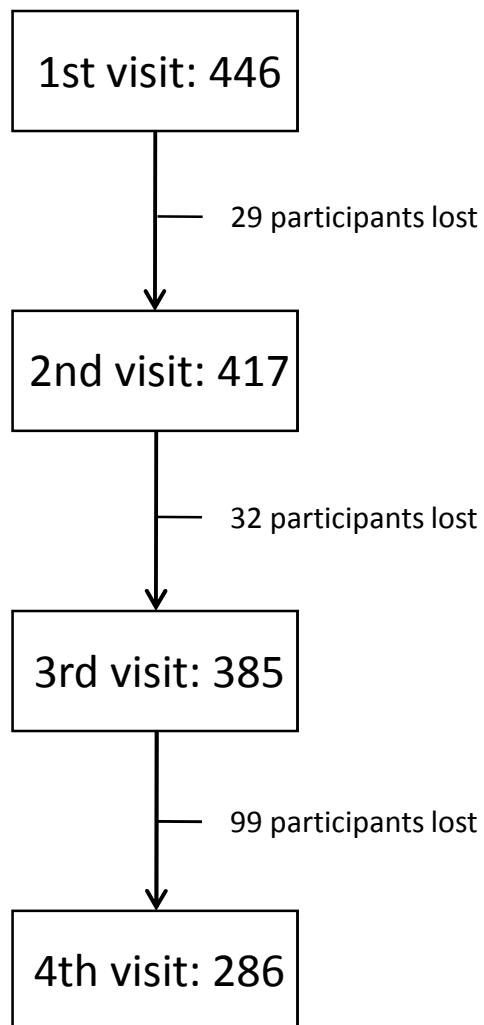

**Figure S1:** Study visit Flowchart

Supplement: Supplementary file 1 — Additional file 1: Figure S1. Study Flowchart. [file 12879_2021_6208_MOESM1_ESM.pdf]
