# Supplementary material for: Longitudinal assessment of SARS-CoV-2 IgG seroconversionamong front-line healthcare workers during the first wave of the Covid-19 pandemic at a tertiary-care hospital in Chile
Source: BMC Infect Dis. 2021 May 26;21:478. doi: 10.1186/s12879-021-06208-2 (PMC8149923; doi:10.1186/s12879-021-06208-2)
Supplement: Supplementary file 2 — Additional file 2: Figure S2. Comparison of IgG ratios of RT-PCR positive vs. negative participants (line and bars represent mean with 95% CI)(p = 0.09, t-test). [file 12879_2021_6208_MOESM2_ESM.pdf]

# FIGURE S2.

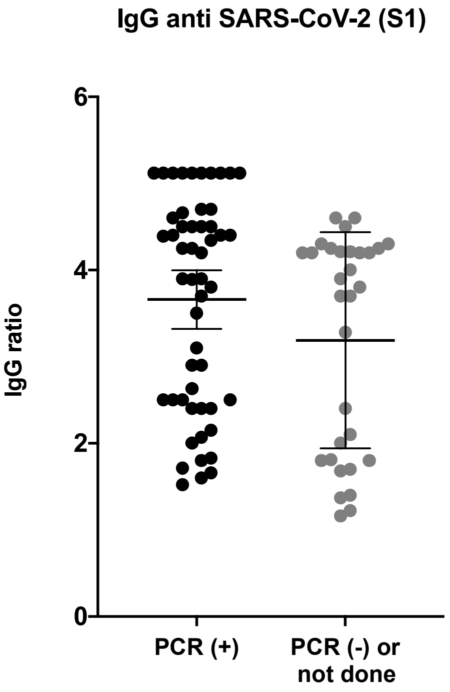


**Figure S2:** Comparison of IgG ratios of RT-PCR positive vs. negative participants (line and bars represent mean with 95% CI) (*p*=0.09, t-test).
