# Supplementary material for: Longitudinal assessment of SARS-CoV-2 IgG seroconversionamong front-line healthcare workers during the first wave of the Covid-19 pandemic at a tertiary-care hospital in Chile
Source: BMC Infect Dis. 2021 May 26;21:478. doi: 10.1186/s12879-021-06208-2 (PMC8149923; doi:10.1186/s12879-021-06208-2)
Supplement: Supplementary file 3 — Additional file 3: Table S1. Demographic variables of high-risk and low-risk groups. [file 12879_2021_6208_MOESM3_ESM.docx]

**Table S1.Demographic variables of high-risk and low-risk groups.**

| Variables | **Total**  **n=446** | **High Risk**  **n=412** | **Low Risk**  **n=34** | ***p* value** |
| --- | --- | --- | --- | --- |
| Female  Male | 324 (72.6)  122 (27.4) | 309 (75.0)  103 (25.0) | 15 (44.1)  19 (55.9) | 0.000 |
| Median age (years) | 39 (21-67) | 39 (21-67) | 45 (27-63) | 0.001 |
| Profession |  |  |  |  |
| Physician | 163 (36.5) | 146 (35.4) | 17 (50.0) | 0.090 |
| Nurse | 139 (31.2) | 132 (32.0) | 7 (20.6) | 0.166 |
| Medical technician | 11 (2.5) | 6 (1.5) | 5 (14.7) | 0.000 |
| Respiratory therapist | 24 (5.4) | 24 (5.8) | - | 0.148 |
| Paramedic technician | 104 (23.3) | 101 (24.5) | 3 (8.8) | 0.038 |
| Administrative worker | 5 (1.1) | 3 (0.7) | 2 (5.9) | 0.006 |
| Comorbidities |  |  |  |  |
| None | 256 (57.4) | 239 (58.0) | 17 (50.0) | 0.364 |
| Diabetes | 4 (0.9) | 4 (1.0) | - | 0.564 |
| Hypertension | 29 (6.5) | 22 (5.3) | 7 (20.6) | 0.001 |
| Asthma | 17 (3.8) | 17 (4.1) | - | 0.227 |
| Obesity | 31 (7.0) | 30 (7.3) | 1 (2.9) | 0.339 |
| Smoker | 91 (20.4) | 85 (20.6) | 6 (17.6) | 0.678 |
| Other | 132 (29.6) | 122 (29.6) | 10 (29.4) | 0.980 |
