# Supplementary material for: Longitudinal assessment of SARS-CoV-2 IgG seroconversionamong front-line healthcare workers during the first wave of the Covid-19 pandemic at a tertiary-care hospital in Chile
Source: BMC Infect Dis. 2021 May 26;21:478. doi: 10.1186/s12879-021-06208-2 (PMC8149923; doi:10.1186/s12879-021-06208-2)
Supplement: Supplementary file 4 — Additional file 4: Table S2. Characteristics of seropositive and seronegative healthcare workers. [file 12879_2021_6208_MOESM4_ESM.docx]

**Table S2. Characteristics of seropositive and seronegative healthcare workers.**

| **Variables** | **Total**  **n =446**  **(%)** | **Seronegative**  **n = 339**  **(%)** | **Seropositive**  **n = 107**  **(%)** | ***p* value** |
| --- | --- | --- | --- | --- |
| Gender; Female | 324 (72.6) | 240 (70.8) | 84 (78.5) | 0.12 |
| Age, years (median and range) | 39 (21-67) | 39.6 (21-67) | 37.8 (23-65) | 0.09 |
| Work area  High-risk for Covid-19  Low-risk for Covid-19 | 412 (92.4)  34(7.6) | 312 (92.1)  27(7.9) | 100 (93.5)  7(6.5) | 0.63 |
| Comorbidities & medications | | | | |
| Diabetes | 4 (0.9) | 2 (0.6) | 2 (1.9) | 0.22 |
| Hypertension | 29 (6.5) | 23 (6.8) | 6 (5.6) | 0.67 |
| Obesity | 31 (7.0) | 26 (7.7) | 5 (4.7) | 0.29 |
| Asthma | 17 (3.8) | 13 (3.8) | 4 (3.7) | 0.96 |
| Current smoker | 91 (20.4) | 79 (23.3) | 12 (11.2) | 0.007 |
| No comorbidities | 256 (57.4) | 203 (59.9) | 53 (49.5) | 0.06 |
| Use of ACE inhibitors | 25 (5.6) | 20 (5.9) | 5 (4.7) | 0.63 |
| Epidemiological risk factors | | | | |
| Non-occupational Covid-19 contact | 30 (6.7) | 20 (5.9) | 10 (9.3) | 0.22 |
| International travel (previous 3 months) | 128 (28.7) | 88 (26) | 40 (37.4) | 0.023 |
| Covid-19 related symptoms within 4 weeks of serological testing | | | | |
| At least one | 190 (42.6) | 129 (38.1) | 61 (57) | 0.001 |
| None | 256 (57.4) | 210 (61.0) | 46 (43.0) | 0.001 |
| Fever | 26 (5.8) | 5 (1.5) | 21 (19.6) | 0.000 |
| Cough | 60 (13.5) | 32 (9.4) | 28 (26.2) | 0.000 |
| Anosmia + Ageusia | 53 (11.9) | 5 (1.5) | 48 (44.9) | 0.000 |
| Odynophagia (sore throat) | 77 (17.3) | 50(14.7) | 27 (25.2) | 0.012 |
| Coryza | 44 (9.9) | 30 (8.8) | 14 (13.1) | 0.200 |
| Myalgia | 48 (10.8) | 21 (6.2) | 27 (25.2) | 0.000 |
| Abdominal pain | 7 (1.6) | 4 (1.2) | 3 (2.8) | 0.239 |
| Nausea + Vomiting | 28 (6.3) | 7 (25.0) | 21 (19.6) | 0.000 |
| Chest pain | 24 (5.4) | 8 (2.4) | 16 (15.0) | 0.000 |
