# Supplementary material for: Longitudinal assessment of SARS-CoV-2 IgG seroconversionamong front-line healthcare workers during the first wave of the Covid-19 pandemic at a tertiary-care hospital in Chile
Source: BMC Infect Dis. 2021 May 26;21:478. doi: 10.1186/s12879-021-06208-2 (PMC8149923; doi:10.1186/s12879-021-06208-2)
Supplement: Supplementary file 5 — Additional file 5. Participant Questionnaire. [file 12879_2021_6208_MOESM5_ESM.pdf]

Participant Questionnaire

(Translated from original Spanish version)

Study ID:__________

Date of registry:____________

Demographic Data

Name:

RUT (ID N°):

Date of birth:

Age:

Gender:

e-mail:

Medical history

Have you been diagnosed with one of the following diseases? Mark all that apply

- None
- Diabetes
- Hypertension
- Chronic Heart Failure
- Chronic Kidney Failure
- Pulmonary disease
- Asthma
- Obesity
- Oncologic disease
- Transplant
- Other

If other, please specify:

Are you currently smoking?

- Yes
- No

If yes, number of cigarettes/day:

- 0-5
- 6-10
- 10-20
- >20

Are you currently using any medication?

- Yes
- No

If yes, please describe the type of medication:

- ACE inhibitors
- Anti-inflammatory drugs (NSAIDs)
- Hydroxychloroquine
- Steroids
- Immunosuppressants
- Other, please specify:

Type of immunosuppressants:

- Azathioprine
- Cyclosporine
- Methothrexate
- Mycophenolate mofetil
- Other, please specify:

Job position

Profession

- Technician
- Nurse
- Physician
- Respiratory therapist
- Administrative

Work place (Unit):

- ICU
- Stepdown unit
- Medical ward
- Emergency department
- Non-Covid ICU: coronary ICU

Do you work in another hospital?

- Yes
- No

If yes, in what center and occupation:

Covid-19 risk evaluation

Have you been diagnosed with Covid-19 at the moment of filling this survey?

- Yes
- No

If yes, please specify date:

Have you been in close contact with Covid-19 confirmed or probable patients outside de hospital?

- Yes
- No

If yes, when were you contact of a covid-19 patient:

  - 3 weeks
  - 2-3 weeks
  - 1-2 weeks
  - Don’t remember

Have you presented any of the following symptoms during the last month (mark all that apply):

- None
- Fever
- Cough
- Malaise
- Myalgia
- Anosmia or ageusia
- Chest pain

- Sore throat
- Abdominal pain
- Nausea
- Nasal congestion
- Dyspnea
- Skin rash
- Headache
- Conjunctivitis
- Diarrhea

Have you been tested with Covid-19 RT-PCR?

- Yes
- No

If yes, the reason why you were tested:

- Close Contact
- Covid-19 symptoms
- Travel

Date of test and result:

Have you travelled outside Chile in the last 3 months?

- Yes
- No

If yes, countries you visited:

- China
- Korea
- Japan
- Thailand
- Italy
- France
- Spain
- USA
- Other, please specify:

Date of travel:

Follow up questionnaire

(For visits 2, 3 and 4)

Have you been diagnosed with Covid-19 at the moment of filling this survey?

- No
- Yes

If yes, please specify date:

Have you been in close contact with Covid-19 confirmed or probable patients outside de hospital?

- Yes
- No

If yes, when were you contact of a Covid-19 patient

  - 3 weeks
  - 2-3 weeks
  - 1-2 weeks
  - Don't remember

Have you presented any of the following symptoms during the last month (mark all that apply):

- None
- Fever
- Cough
- Malaise
- Myalgia
- Anosmia or ageusia
- Chest pain
- Sore throat
- Abdominal pain
- Nausea
- Nasal congestion
- Dyspnea
- Skin rash
- Headache
- Conjunctivitis
- Diarrhea

Have you been tested with Covid-19 RT-PCR?

- No
- Yes

If yes, the reason why you were tested:

- Close Contact
- Covid-19 symptoms
- Travel

Date of test and result:
